# Patient‐Specific Biases in Fat Fraction Estimates of Malignant Bone Marrow due to Relaxation Times Measured With STEAM at 3T

**DOI:** 10.1002/nbm.70242

**Published:** 2026-02-24

**Authors:** Yassine N. Azma, David J. Collins, Pete J. Lally, Nina Tunariu, Dow‐Mu Koh, Christina Messiou, Geoff Charles‐Edwards, Christina Triantafyllou, Neal K. Bangerter, Jessica M. Winfield

**Affiliations:** ^1^ MRI Unit Royal Marsden NHS Foundation Trust London UK; ^2^ Department of Bioengineering Imperial College London London UK; ^3^ Division of Radiotherapy and Imaging The Institute of Cancer Research London UK; ^4^ Research and Collaborations GBI Siemens Healthcare Ltd Camberley UK; ^5^ Department of Electrical and Computer Engineering Boise State College of Engineering Boise Idaho USA

**Keywords:** bone marrow, fat quantification, metastasis, relaxometry, spectroscopy, whole‐body

## Abstract

Semiquantitative fat fraction estimation using 2‐point Dixon sequences is widely used in whole‐body (WB) MR imaging for malignant bone disease but is biased by relaxation times. Understanding this bias requires water‐ and fat‐specific relaxometry data in normal‐appearing marrow and lesions. This study measured bone marrow relaxation times in healthy volunteers and WB‐MRI patients using MRS at 3T. Five healthy female volunteers (mean age 38.0 ± 2.5 years) and 24 patients with malignant bone disease undergoing clinical WB‐MRI (13 male; mean age 67.7 ± 9.7 years; primary cancers: breast = 5, melanoma = 1, multiple myeloma = 8, prostate = 10) underwent variable inversion/echo time STEAM and 3D gradient echo fat‐water imaging. MRS water and fat peaks were fitted to determine T1, T2, R2* (from linewidths) and proton density fat fraction (PDFF). Scan‐rescan repeatability of MRS parameters was assessed in volunteers. Lesions were classified by disease state according to clinical reports and segmented in Dixon imaging data for comparison of fat fraction estimates with MRS. Repeatability was evaluated using coefficients of variation. Summary statistics (mean, standard deviation and range) were reported; exploratory inferential statistics were also determined with normality (Shapiro–Wilk) and variance (Levene's) tests before one‐way ANOVA and Tukey's comparisons (*p* < 0.05). Monte–Carlo simulations assessed relaxation bias on PDFF. All quantitative MRS parameters were repeatable (coefficient of variation < 10%). Water T1 and T2 were most sensitive to disease state in patients, ranging from 1121 to 2206 and from 15 to 71 ms respectively, and were demonstrated to substantially affect 2‐point Dixon fat fraction estimates with Monte–Carlo simulation. Imaging PDFF achieves closer agreement with MRS PDFF than 2‐point Dixon methods. These findings remain preliminary due to the small sample size, but they suggest value in future studies with larger cohorts evaluating water relaxation times or PDFF in malignant bone disease.

## Graphical abstract

Water relaxation times measured with variable‐inversion and echo time STEAM show sensitivity to disease state in bone lesions.

In a volunteer cohort, these measurements are repeatable on a per‐subject basis in both L5 and the femoral head (average CoV < 10%).

Despite the small sample size, significant impacts on fat fraction estimation due to relaxation times using 2‐point T1w‐Dixon imaging recommended by international guidelines for whole‐body MRI.

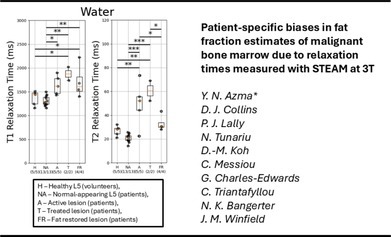

**Abbreviations**
ADC: apparent diffusion coefficient
FF: signal‐weighted fat fraction
PD: proton density
PDFF: proton density fat fraction
R2*: transverse relaxation rate
STEAM: stimulated echo acquisition mode
WB‐MRI: whole‐body MRI

## 1 Introduction

Whole‐body MRI (WB‐MRI) has become essential in the evaluation of skeletal bone disease and is recommended in international guidelines for use in advanced prostate cancer [1], multiple myeloma [2] and cancer screening [3]. Studies have demonstrated the sensitivity of the apparent diffusion coefficient (ADC) and signal‐weighted fat fraction (FF) for lesion characterization and response assessment [4, 5, 6, 7].

Despite the successes of the FF for determining treatment response, it is confounded by a number of biases compared to the proton density fat fraction (PDFF). These biases are due to T1 relaxation [8], T2* decay [9], noise [10], the spectral complexity of fat [11], field inhomogeneity [12] and readout scheme [13]. Owing to the length of a WB‐MRI protocol, a 2‐point T1‐weighted Dixon acquisition is recommended as a combined source of morphological imaging and a semiquantitative measure of FF in MET‐RADS‐P [1] and MY‐RADS [2]. These are two standardized frameworks for the acquisition and reporting of imaging data in malignant bone disease in advanced prostate cancer and multiple myeloma, respectively.

T1 and T2 relaxation times in vertebral bone marrow have been characterized in healthy subjects in previous studies using both MRS and imaging [14, 15]. However, there are limited data assessing relaxation times in bone metastases with MRS, which unambiguously separates fat and water [16]. Owing to the relaxation time biases of the FF, it is necessary to measure these relaxation times to provide clarity about the severity of this bias and how it changes with disease state. Given the heterogeneity of bone metastases, it can be assumed that the impact of this bias varies considerably both within and between subjects [17].

The aim of this study was to assess whether bone marrow relaxation times are patient‐specific in malignant bone disease and to evaluate the implications of this variability for imaging‐based fat quantification.

## 2 Experimental

This study was approved by a national research ethics committee (NCT05118555, http://clinicaltrials.gov). All patients provided verbal consent for the acquisition of additional research data as part of routine clinical examinations, and all healthy volunteers provided written consent to participate in the study.

### 2.1 Healthy Volunteers

Female healthy volunteers were recruited for the study to assess repeatability of MRS relaxometry in bone marrow as they have more hematopoietic marrow than males [18]. The lower fat content in the female bone marrow of the axial skeleton results in a closer mimic to active or treated bone lesions, which have low fat content [7].

MRS data were acquired in the femoral head and the fifth lumbar vertebra (L5). The femoral head was chosen as a surrogate for sclerotic bone disease as the head is both fatty but also highly trabeculated [19]. The repeatability measurements between these two sites should be close to a ceiling for the repeatability of fatty marrow in the patient population as well, which will likely have greater fat content due to age [20].

After both anatomical sites were scanned, the volunteer exited the scanner for a short period (~2 min) before the entire process was repeated. This included the volunteer leaving the table before repositioning coils and re‐shimming.

### 2.2 Patients

Patients scheduled for WB‐MRI examinations were prospectively assessed based on prior imaging for suitability in this study. The WB‐MRI service is oriented towards disease evaluation and response assessment for patients with castration‐resistant prostate cancer, breast cancer and multiple myeloma, although other cancers may also be indicated for WB‐MRI by clinicians. No restrictions were made at the point of recruitment with regards to therapeutic history, so prior treatments include and are not limited to systemic therapies and autologous stem cell transplants.

Following completion of the routine clinical WB examination, MRS voxels were placed in the largest focal lesion of at least 1.5‐cm diameter in‐plane on routine imaging in the pelvis. In the case of there being no evidence of focal bone disease between the femoral heads and L5, data were taken from L5. In patients, data taken from L5 were referred to as normal‐appearing. This definition contrasts with healthy marrow from volunteers to account for the potential presence of subclinical bone disease or effects of prior treatment.

### 2.3 Acquisition

All scanning was performed on a 3T MR system (MAGNETOM Vida, Siemens Healthineers, Forchheim, Germany) using a 32‐channel spine receiver coil and 18‐channel anterior body array receiver coil. Localization of spectroscopy voxels was performed using the fat‐only images of a 3D T1‐weighted gradient echo Dixon sequence acquired in the transverse plane and reformatted in sagittal and coronal planes.

STEAM was used for acquiring MRS data in order to minimize the impact of scalar coupling [21]. For measuring T1, an in‐house STEAM pulse sequence with a hyperbolic secant pulse for inversion was used. Considerations about the selection of RF pulses used in this study can be found in Supplementary Materials Figures 1–5. T2 measurement was performed with variable echo time measurements. Further details regarding the MRS acquisition protocol can be found in Table 1 below.

**TABLE 1:** Spectroscopy acquisition parameters.

MRS relaxometry		
	Variable echo time STEAM (T2 estimation)	Variable inversion time STEAM (T1 estimation)
Echo time (ms)	20, 25, 30, 35, 40, 50, 60, 80, 100	20
Inversion time (ms)	N/A	15, 70, 150, 300, 500, 1000, 2500, 4000
Mixing time (ms)	10	
Repetition time (ms)	3500	5000
Number of averages	6	
Voxel size (mm|AP × RL × FH)	15 × 15 × 15 in lesions 20 × 20 × 15 in L5 20 × 20 × 20 in femoral head	
Matrix	2048	
Receive bandwidth (Hz)	2400	
Delta frequency (ppm)	−2.3^a^	
Preparation scans	4	
Time per contrast (min:ss)	00:35	00:50

^a^ Excitation offset relative to water/centre frequency.

Axial Dixon imaging was performed based on our institution's routine WB‐MRI Dixon protocol, which is as follows: TE1/TE2 = 2.46/3.69 ms, TR = 7.14 ms, FA = 20°, FOV = 430 × 323 × 220 mm^3^, matrix = [256 × 192 × 44], acceleration = 2 × 2 CAIPIRINHA, acquisition time = 17 s. The manufacturer's multiecho PDFF imaging sequence, from the LiverLab option, which uses a hybrid fitting approach was also used for a quantitative comparison of PDFF and R2* [22]. Parameters were as follows: TE1/ΔTE/TE6 = 1.13/1.13/6.78 ms, TR = 8.7 ms, FA = 4°, FOV = 430 × 323 × 220 mm^3^, matrix = [256 × 192 × 44], acceleration = 2 × 2 CAIPIRINHA, acquisition time = 18 s. All data were acquired with the central slice of the imaging volume going through the lesion (or L5 where appropriate) in free breathing. Slice positions were matched for both imaging sequences with the clinical axial echo‐planar diffusion‐weighted imaging sequence, which had the following parameters: TE = 55 ms, TR = 6300 ms, STIR fat suppression (TI = 240 ms), FOV = 430 × 347 × 200 mm^3^, matrix = [134 × 108 × 40], acceleration = 2× GRAPPA, monopolar diffusion encoding gradients, *b* values = 50, 600, 900 s/mm^2^ with 3, 3 and 6 averages, respectively, acquisition time = 4 min 7 s.

### 2.4 Processing

Preprocessing and fitting were performed in Python 3.12. Preprocessing was performed using the SUSPECT library (available on Github: https://github.com/openmrslab/suspect), which involved channel combination with whitened singular value decomposition [23] and frequency alignment with spectral registration in the time domain [24]. Fitting was performed using an in‐house frequency‐domain fitting routine with a 10‐peak fat model [25]. Water and fat peaks were assumed to have pseudo‐Voigt line‐shapes [26]. Water peaks were assumed to be 0.5 × Lorentzian, 0.5 × Gaussian as a compromise for potential sclerotic changes to the bone in the patient population. Fat peaks were assumed to be 0.2 Lorentzian, 0.8 Gaussian as in Dieckmeyer et al. [27]. A fixed chemical shift frequency relationship between all fat peaks was presumed resulting in only methyl and water frequencies being allowed to vary. Constraints on fat amplitudes were included in accordance with the model of a generic triglyceride introduced by Hamilton et al. [25].

Alpha‐olefin (2.03 ppm) and alpha‐carboxyl (2.25 ppm), referred to as composite from hereon, were modelled to share a relaxation time, which was deemed to be a reasonable assumption based on literature values in adipose tissue [28]. Five fat peaks (methyl 0.9 ppm, methylene 1.3 ppm, composite 2.25 ppm, diacyl 2.77 ppm and olefin 5.31 ppm) and water were modelled as having unique T1 values. Methylene, composite and water were modelled as having unique T2 values. In the case of T1 and T2 estimations, methylene was used as a common relaxation time for fat peaks that were not modelled separately. Water and methylene were modelled with separate linewidths, whilst all remaining fat peaks shared a linewidth. The inversion efficiency was assumed to be equal for all resonances in line with Bloch simulation of the inversion pulse's performance with off resonance in Supplementary Materials Figure 2.

The first step involved fitting the TE = 20 ms spectrum to obtain initial guesses of peak amplitudes for later fits. For the five healthy female volunteers, their L5 vertebrae were used to determine a common number of double bonds and number of methylene‐interrupted double bonds in a similar fashion to Dieckmeyer et al. [27]. This was done as a means of providing initial guesses for the glycerol and olefin amplitudes in the later joint multi‐TE and multi‐TI fitting. This assumed fatty acid composition was also used in the patients, owing to the healthy volunteers being a homogeneous cohort. The patient cohort had bone marrow that varied significantly in fat content, age, sex, disease state and therapeutic history, making any population‐wide measure unsuitable. It was assumed that this would have minimal impact on the resultant PDFF.

Owing to the use of a long‐TE for the possibility of osteolytic bone disease, which is predominantly water‐like, J‐coupling can result in inaccuracies in both amplitudes and T2 relaxation times, leading to errors in the subsequent calculation of PDFF [21]. Joint fitting of the multi‐TE data was performed twice: first using a subset of echo times (20 to 50 ms), and then using all echo times to assess this effect. PDFF was then calculated from the amplitudes as normal.

The multi‐TI data were fitted to acquire T1 by first assuming all peaks were inverted for the first inversion time and upright for the last. To minimise the confounding effect of zero‐crossings, the data were then fitted with sequential subsets, still including the last inversion time, and the newly added spectrum to the joint fitting was fitted in its pre‐processed state and with an additional 180° rotation. The fit with the lower cost decided the sign of the recently added spectrum before including the next. This was repeated until all inversion times were included. The signal models used are shown below in Equations (1) and (2).

(1) $$S\left(\mathrm{TE}\right)=\sum_{n}A_{0,n}\left(\mathrm{LS}F_{n}\times L\left(f_{0,n},{\mathrm{LW}}_{n}\right)+\left(1-\mathrm{LS}F_{n}\right)\times G\left(f_{0,n},{\mathrm{LW}}_{n}\right)\right)e^{-\frac{\mathrm{TE}}{T_{2,n}}}$$

where *A*_0_ is the T2‐corrected amplitude (taken to be the proton density), *TE* is the echo time, LSF is the Lorentzian shape fraction, L is the Lorentzian function in the frequency‐domain, G is the Gaussian function in the frequency‐domain, and T2 is the transverse relaxation time.

(2) $$\begin{array}{c} \begin{array}{c} S\left(\mathrm{TI}\right)=\sum_{n}A_{0,n}\left(\mathrm{LS}F_{n}\times L\left(f_{0,n},{\mathrm{LW}}_{n}\right)+\left(1-\mathrm{LS}F_{n}\right)\times G\left(f_{0,n},{\mathrm{LW}}_{n}\right)\right)\left(1-2\beta e^{-\frac{\mathrm{TI}}{T_{1,n}}}+e^{-\frac{\mathrm{TR}}{T_{1}}}\right) \end{array} \end{array}$$

where *A*_0_ is the amplitude following complete longitudinal relaxation, *β* is the inversion efficiency, TI is the inversion time, T1 is the longitudinal relaxation time, and TR is the repetition time.

The PDFF was calculated as follows:

(3) $$\begin{array}{c} \text{PDFF}=\frac{\sum_{n}F_{0,n}}{W_{0}+\sum_{n}F_{0,n}} \end{array}$$

where *F*_0,n_ is the T2‐corrected amplitude of the nth fat peak included in the model described earlier in the text, and *W*_0_ is the T2‐corrected amplitude of the water peak.

Linewidths of the pseudo‐Voigt peaks obtained from the joint fitting of the multi‐TE data were converted into R2* using the following relation:

(4) $${R_{2}}^{*}=\pi LW$$

where R2* is the transverse relaxation rate and *LW* is the spectral linewidth.

The T1 and R2* relaxation times from each disease state in bone marrow were used for Monte–Carlo simulations of the RF‐spoiled gradient echo signal in idealized conditions (assumptions of no field inhomogeneity and perfect spoiling) with additive complex white Gaussian noise. Fat fraction estimates were calculated using the same echo times as the 2‐point Dixon mentioned previously with both PD‐ and T1‐weighting. Signals were generated with a nine‐peak model of bone marrow fat [29], the naïve assumption of a single fat peak and the assumption of both a single fat peak and no R2* decay. This combination of simulations was performed to examine the compounding effects of noise, T1, R2* and spectral model effects for 2‐point FF estimates.

### 2.5 Analysis

T1 and T2 were reported for each modelled resonance. R2* was reported for the methylene and water resonances. MRS‐derived PDFF was calculated for all volunteer and patient data. Relaxation times were not reported if the relative Cramer‐Rao lower bound exceeded 25% or the peak amplitude was less than 1% of the largest peak amplitude (water or methylene).

Bone lesions in patients were classified based on clinical reports made by radiologists (N.T. and C.M., > 10 years of experience each). Active disease was represented by a low ADC and low FF from a T1‐weighted Dixon. Treated disease had high ADC and low FF. Lesions that had been active or treated in previous imaging and now demonstrated a return of the marrow fat were deemed ‘fat restored.’

Bland–Altman analysis was used to assess the repeatability of T1, T2, R2* and PDFF in the volunteer cohort for L5 and the femoral head separately. Ensemble coefficients of variation were obtained following log‐transformation of the data for each parameter using previously reported methods by Sullivan et al. [30] for positive data. All quantitative parameters were tested for equality of variances and normality using the Shapiro–Wilk and Levene's tests, respectively, across disease states. If a parameter was both normal and homoscedastic, one‐way ANOVA was used to test for significant differences in the means amongst all groups. If the null hypothesis was rejected following one‐way ANOVA, Tukey's test was used for pairwise comparisons between all groups.

The entirety of the L5 marrow was outlined in both the T1‐weighted Dixon and PDFF maps using ITK‐SNAP. A single central slice was outlined from the lesion data. Agreement between MRS PDFF and R2* from the water and methylene peaks with MRI methods was assessed with Bland–Altman plots and correlation plots.

## 3 Results

The volunteer cohort consisted of five healthy female volunteers (median age 40 years, range 28–42 years).

Of the 24 patients (11 female, 13 male; median age 68.5 years, range 47–84 years) recruited for the study, 5 had primary breast cancer, 1 had primary melanoma, 8 had multiple myeloma, and 10 had primary prostate cancer. Eleven had focal lesions, 5 of which were active, 2 were treated, and 4 were ‘fat restored.’

**FIGURE 1:**
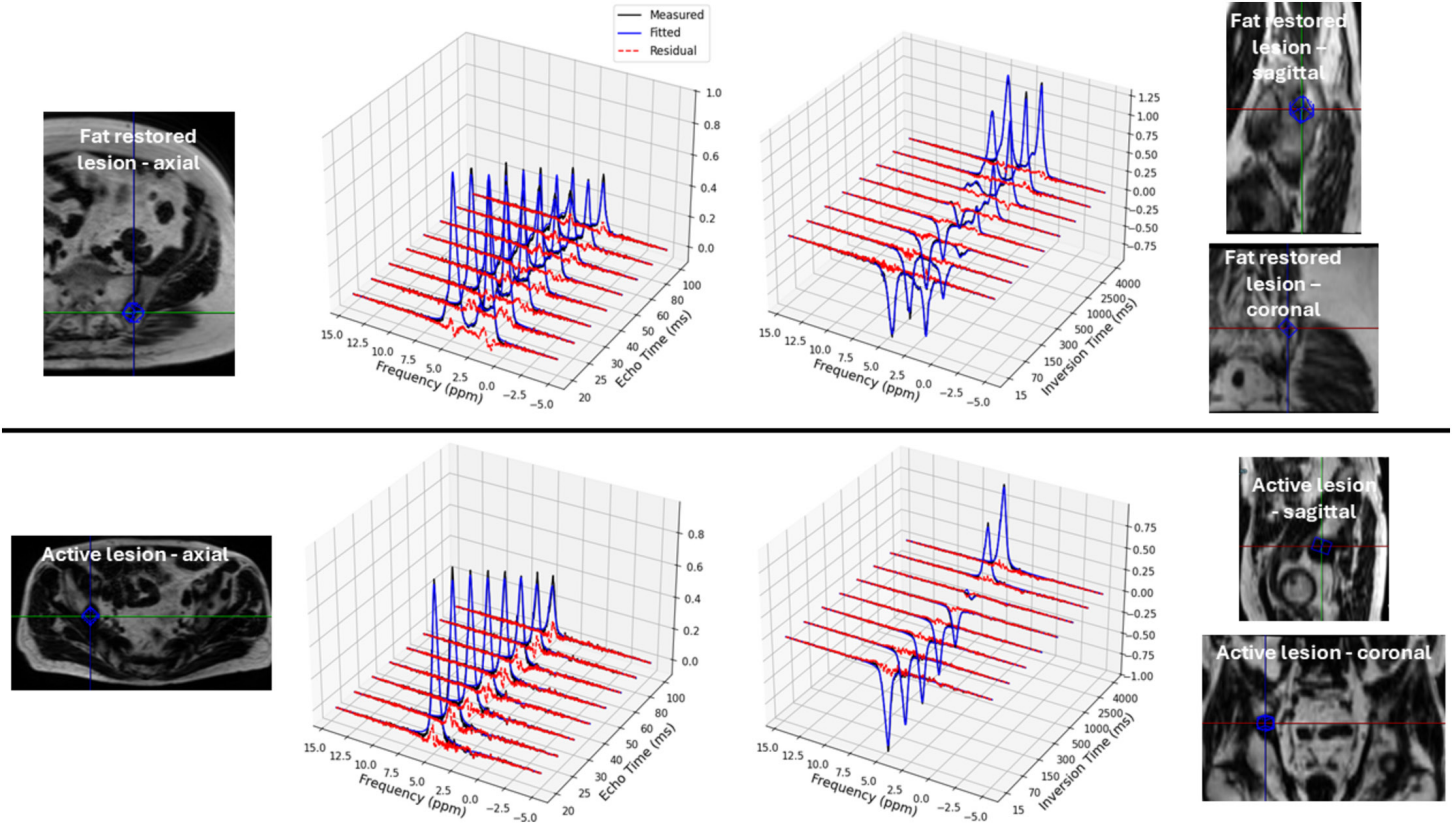
Example variable‐TE and ‐TI spectra from the iliac bones of a fat‐restored lesion and an active lesion from patients with metastatic prostate cancer. Fits and residuals are shown.

Exemplar spectra from iliac bone lesions are shown in Figure 1, along with the chosen voxel location.

**FIGURE 2:**
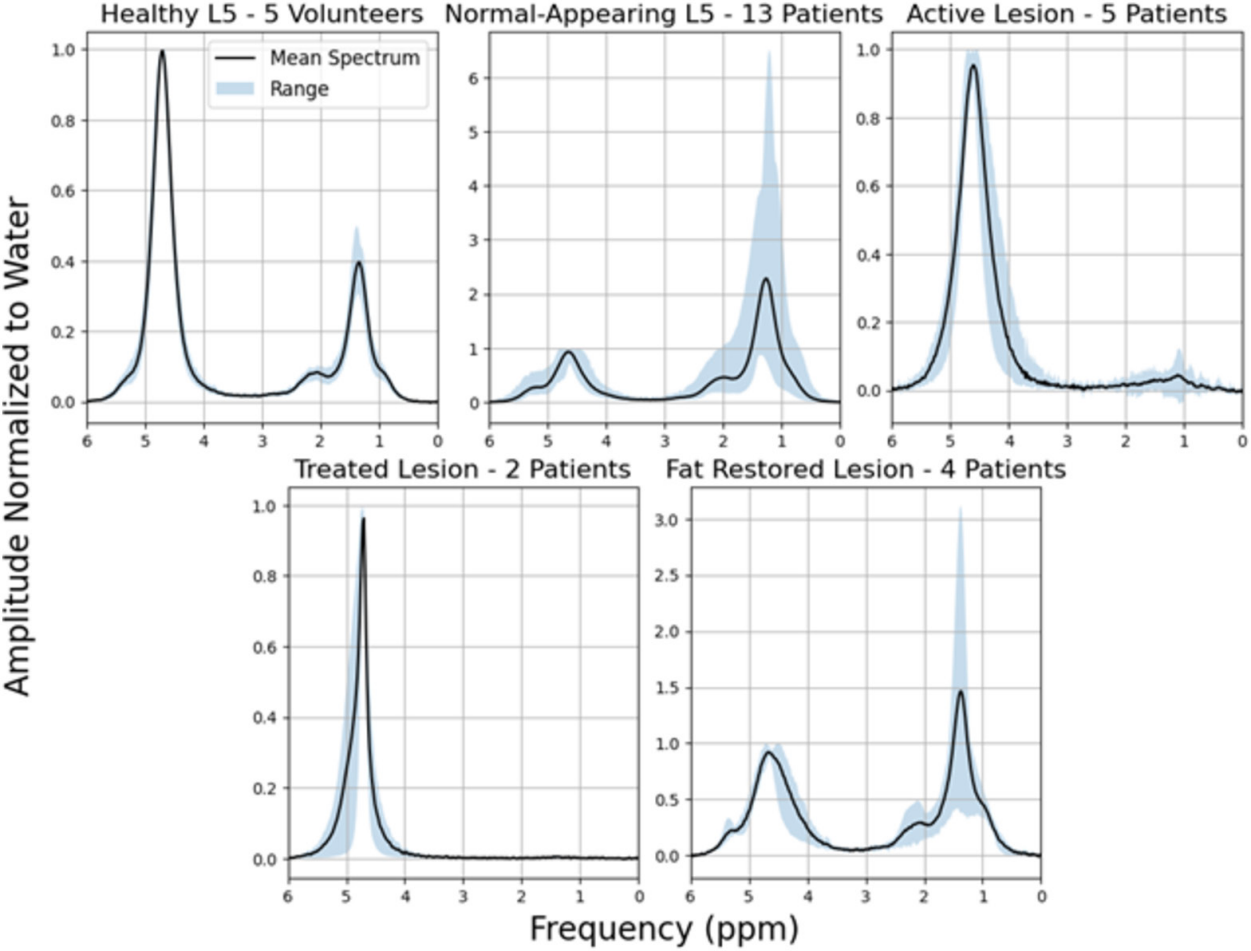
Population average spectrum from TE = 20 ms STEAM (without inversion pulse) for each disease state following baseline subtraction, water peak cantering at 4.7 ppm, and normalization to the water peak height. Blue shading indicates the min‐max range at each frequency across subjects.

Population average spectra are shown in Figure 2. Large variability is seen in the patient population, with both normal‐appearing marrow in L5 and fat‐restored lesions having a wide range of fat content (PDFF from 33.6% to 72.3% and 16.5% to 68.5%, respectively). Average water linewidths in this study were 53 ± 14 Hz (min = 12 Hz, max = 87 Hz). Average inversion efficiency in this study was 0.93 ± 0.03 (min = 0.84, max = 0.98).

**FIGURE 3:**
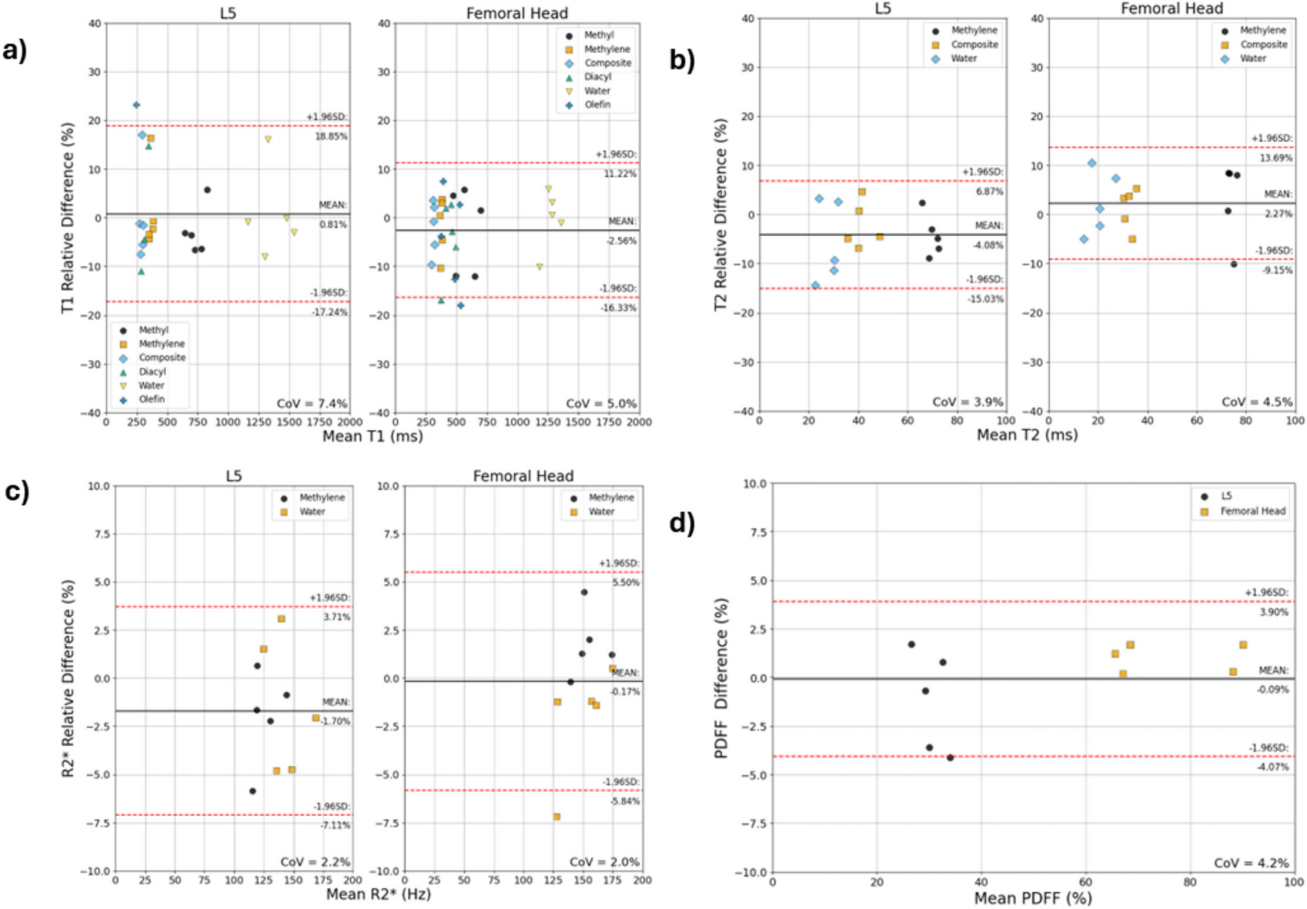
Bland–Altman repeatability analysis of quantitative MRS parameters in L5 and the femoral head of healthy volunteers. (a) T1. (b) T2. (c) R2*. (d) PDFF—note that the y‐axis indicates an arithmetic difference for this subplot.

For the repeatability assessment of T1 measurements, no statistically significant bias between repetitions was seen using a *t* test. T1 and T2 measurement in both sites were highly repeatable (CoV < 8%) (Figure 3a,b).

Coefficients of variation were around 2% for R2* (and by extension the linewidths) in both anatomical sites. A statistically significant bias of 2.1% (*p* < 0.05) was observed between repetitions in the femoral head (Figure 3c). For all repeatability measurements, coefficients of variation per resonance can be found in Supplementary Materials Table 1 and 2 on a pooled and per‐subject average basis, respectively.

PDFF measured using spectroscopy was highly repeatable (CoV = 4.2%) (Figure 3d).

**FIGURE 4:**
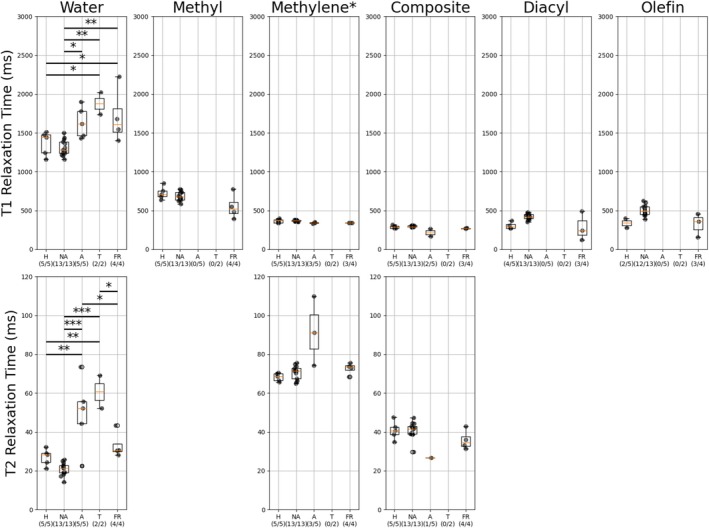
Relaxation times across disease states for each resonance. H—healthy, NA—normal‐appearing, A—active, T—treated and FR—fat restored. X/Y below indicates X patients of Y total had relaxation times that were reported for a particular resonance. *p < 0.05, **p < 0.01, ***p < 0.001. Note that the * next to methylene indicates that it represents all fat peaks without an independent relaxation time shared with methylene.

Significant differences between relaxation times of different resonances by disease state were observed in the water resonance for both T1 and T2 (Figure 4). The T2 of water demonstrated greater sensitivity to different disease states than T1. The T1 and T2 of water varied between 1157–2227 and 14–73 ms, respectively.

**FIGURE 5:**
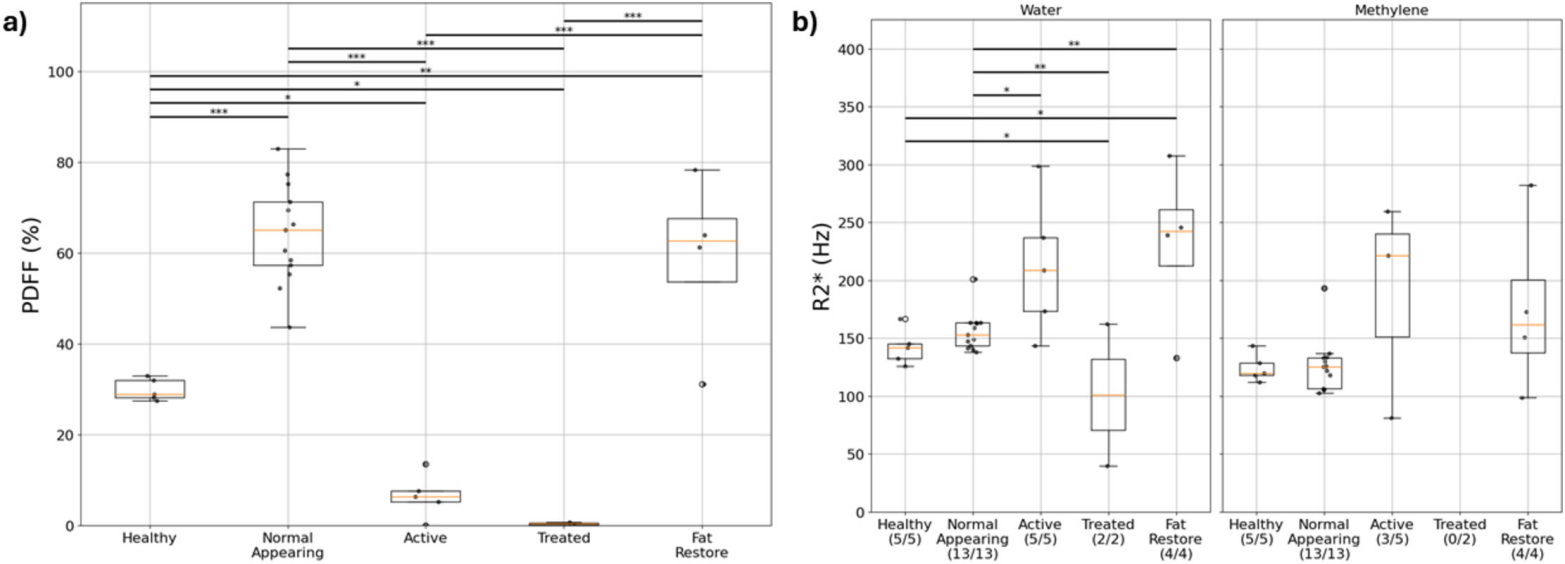
(a) PDFF across disease states. (b) R2* relaxation rates across disease states for water and methylene. *p < 0.05, **p < 0.01, ***p < 0.001.

Proton density FF measured using MRS demonstrated significant differences across multiple disease states (*p* < 0.05). No significant difference in MRS‐PDFF was observed between normal‐appearing and fat‐restored bone lesions (Figure 5a). Significant differences between the R2* of water in multiple disease states were observed (*p* < 0.05). No significant differences were observed for the R2* of methylene (Figure 5b).

The results visualized in Figures 4 and 5 are tabulated in Table 2 with mean values, standard deviations and ranges.

**TABLE 2:** MRS quantitative parameters in volunteers and patients.

Volunteers								
Resonance	Healthy L5 (*n* = 5)				Healthy femoral head (*n* = 5)			
	T1 (ms)	T2 (ms)	R2* (Hz)	PDFF (%)	T1 (ms)	T2 (ms)	R2* (Hz)	PDFF (%)
Methyl (0.9 ppm)	726 ± 74 (635–851)	N/A	N/A	30 ± 2 (27–33)	575 ± 96 (459–704)	N/A	N/A	75 ± 12 (61–91)
Methylene (1.3 ppm)	369 ± 22 (343–398)	68 ± 2 (66–70)	124 ± 11 (112–144)		374 ± 13 (349–391)	74 ± 3 (70–79)	154 ± 12 (139–175)	
Composite (2.25 ppm)	291 ± 19 (270–321)	41 ± 4 (35–47)	N/A		310 ± 13 (280–329)	33 ± 2 (30–37)	N/A	
Diacyl (2.77 ppm)	306 ± 41 (272–372)	N/A	N/A		439 ± 47 (345–506)	N/A	N/A	
Water (4.7 ppm)	1368 ± 140 (1158–1515)	27 ± 4 (21–32)	142 ± 14 (126–167)		1271 ± 69 (1122–1364)	20 ± 5 (14–28)	150 ± 20 (123–175)	
Olefin (5.31 ppm)	340 ± 61 (278–401)	N/A	N/A		460 ± 76 (364–575)	N/A	N/A	

Patients								
	Normal‐appearing L5 (*n* = 13)				Active lesion (*n* = 5)			
Resonance	T1 (ms)	T2 (ms)	R2* (Hz)	PDFF (%)	T1 (ms)	T2 (ms)	R2* (Hz)	PDFF (%)
Methyl (0.9 ppm)	687 ± 57 (585–778)	N/A	N/A	64 ± 11 (44–83)	N/A	N/A	N/A	7 ± 4 (0–14)
Methylene (1.3 ppm)	370 ± 9 (353–384)	71 ± 3 (65–76)	126 ± 23 (102–193)		N/A	92 ± 15 (74–110)	N/A	
Composite (2.25 ppm)	296 ± 9 (282–310)	41 ± 4 (30–47)	N/A		N/A	N/A	N/A	
Diacyl (2.77 ppm)	421 ± 38 (352–481)	N/A	N/A		N/A	N/A	N/A	
Water (4.7 ppm)	1309 ± 100 (1157–1499)	21 ± 3 (14–26)	156 ± 16 (138–201)		1639 ± 181 (1429–1902)	50 ± 17 (22–73)	212 ± 53 (143–299)	
Olefin (5.31 ppm)	503 ± 68 (386–628)	N/A	N/A		N/A	N/A	N/A	

	Treated Lesion (*n* = 2)				Fat Restored Lesion (*n* = 4)			
Resonance	T1 (ms)	T2 (ms)	R2* (Hz)	PDFF (%)	T1 (ms)	T2 (ms)	R2* (Hz)	PDFF (%)
Methyl (0.9 ppm)	N/A	N/A	N/A	0 ± 0 (0–1)	552 ± 140 (395–776)	N/A	N/A	59 ± 17 (31–78)
Methylene (1.3 ppm)	N/A	N/A	N/A		342 ± 1 (341–344)	73 ± 3 (68–76)	176 ± 67 (98–282)	
Composite (2.25 ppm)	N/A	N/A	N/A		272 ± 6 (265–280)	36 ± 4 (31–43)	N/A	
Diacyl (2.77 ppm)	N/A	N/A	N/A		287 ± 155 (121–493)	N/A	N/A	
Water (4.7 ppm)	1880 ± 140 (1740–2020)	61 ± 8 (52–69)	101 ± 61 (40–162)		1714 ± 312 (1404–2228)	33 ± 6 (28–43)	231 ± 63 (133–307)	
Olefin (5.31 ppm)	N/A	N/A	N/A		324 ± 127 (154–459)	N/A	N/A	

**FIGURE 6:**
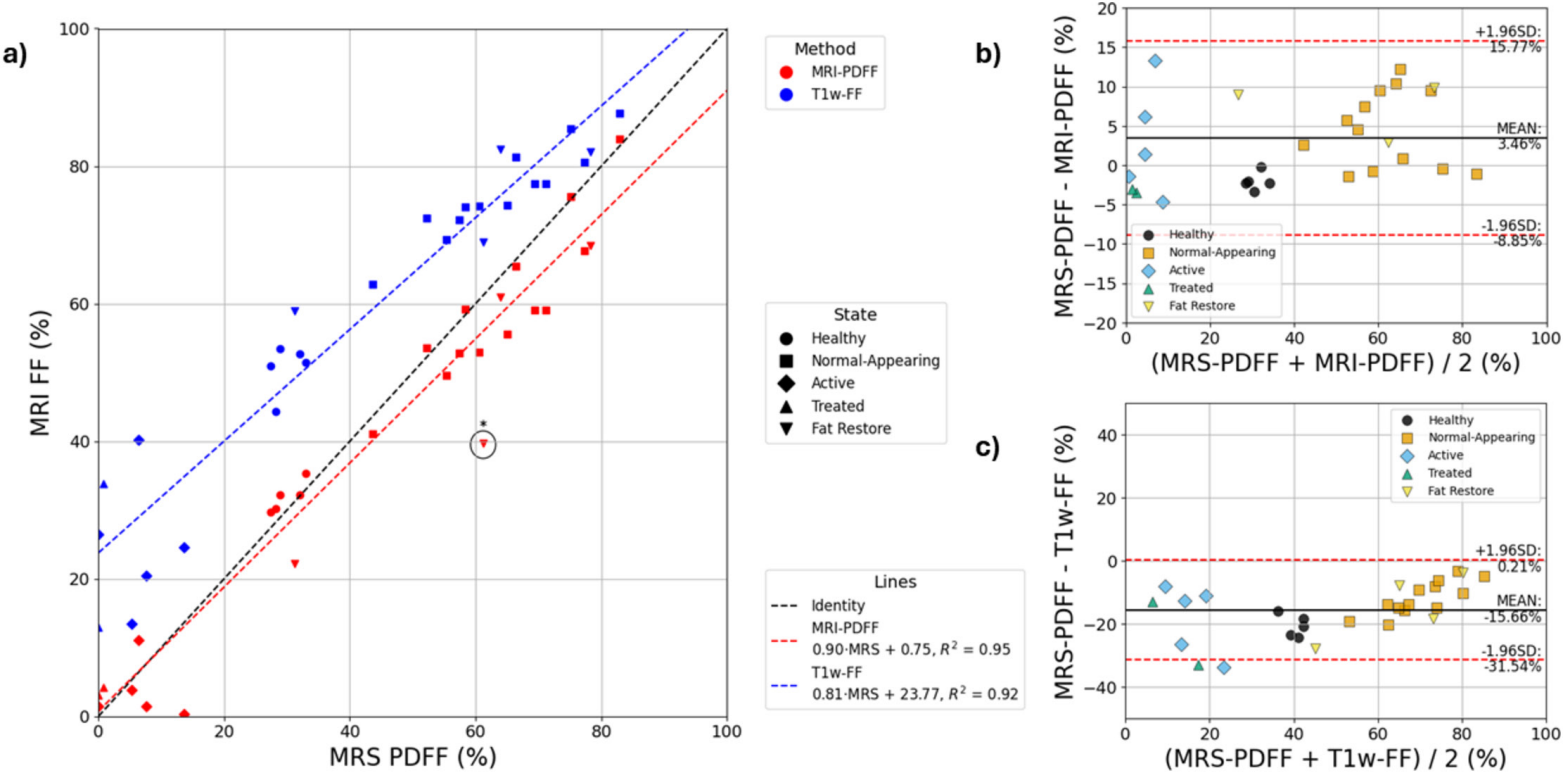
Comparison of PDFF measured with MRS and MRI‐based fat fraction estimates. (a) Correlation plot. Circled data‐point indicates potential error in MRI‐based PDFF due to convergence on a local minimum (fat‐water swap). (b) Bland–Altman comparison between MRS‐PDFF and MRI‐PDFF. (c) Bland–Altman comparison between MRS‐PDFF and T1w‐FF.

**FIGURE 7:**
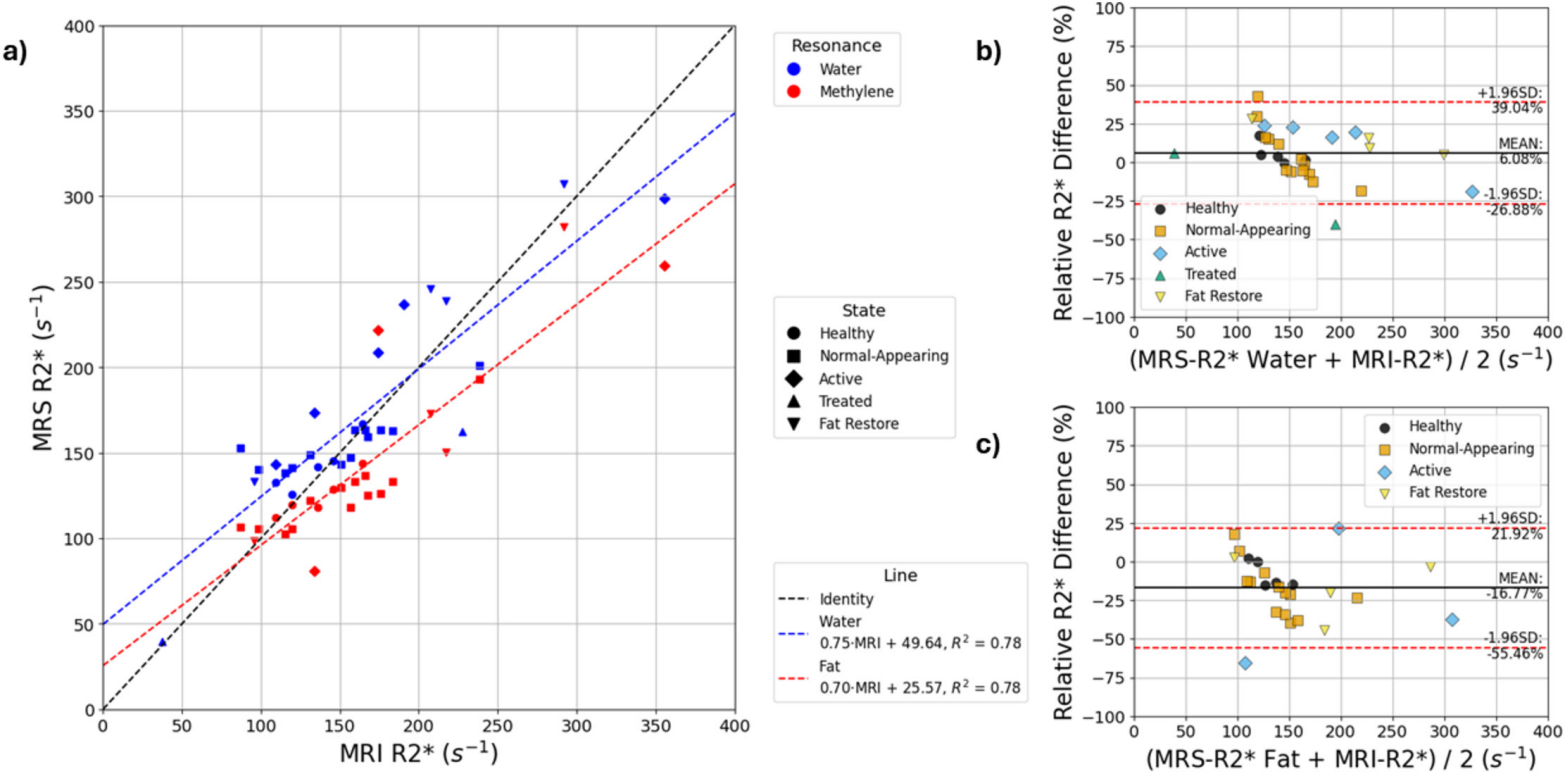
Comparison of R2* measured with MRS and the MRI‐based estimate. (a) Correlation plot. (b) Bland–Altman comparison between R2* extracted from the water peak and the MRI‐R2*. (c) Bland–Altman comparison between R2* extracted from the methylene peak and the MRI‐R2*.

A sublinear relationship between the PDFF from imaging and the MRS‐derived PDFF of 0.92 is observed (Figure 6a,b), although they are well‐correlated (*R* ^2^ = 0.95). Nonlinear bias between the T1‐weighted Dixon and MRS is demonstrated across the patient and volunteer population (average bias = 23.8%) (Figure 6a,c). Large limits of agreement (> ±30%) are seen irrespective of whether the R2* of water or methylene are compared with the effective R2* from imaging, although there is a strong linear relationship between MRI‐based R2* and MRS‐based R2* estimates (*R* ^2^ = 0.78) (Figure 7).

Comparison of T2 and PDFF estimates when fitting the spectra with data from TE = 20 ms to TE = 50 ms and the entirety of the multi‐TE spectra was performed. Estimates were highly correlated for both T2 and PDFF (*R* ^2^ = 0.85 and *R* ^2^ = 1.00, respectively), although the T2 estimates for fat peaks show greater scatter (Supplementary Materials Figures 6 and 7).

**FIGURE 8:**
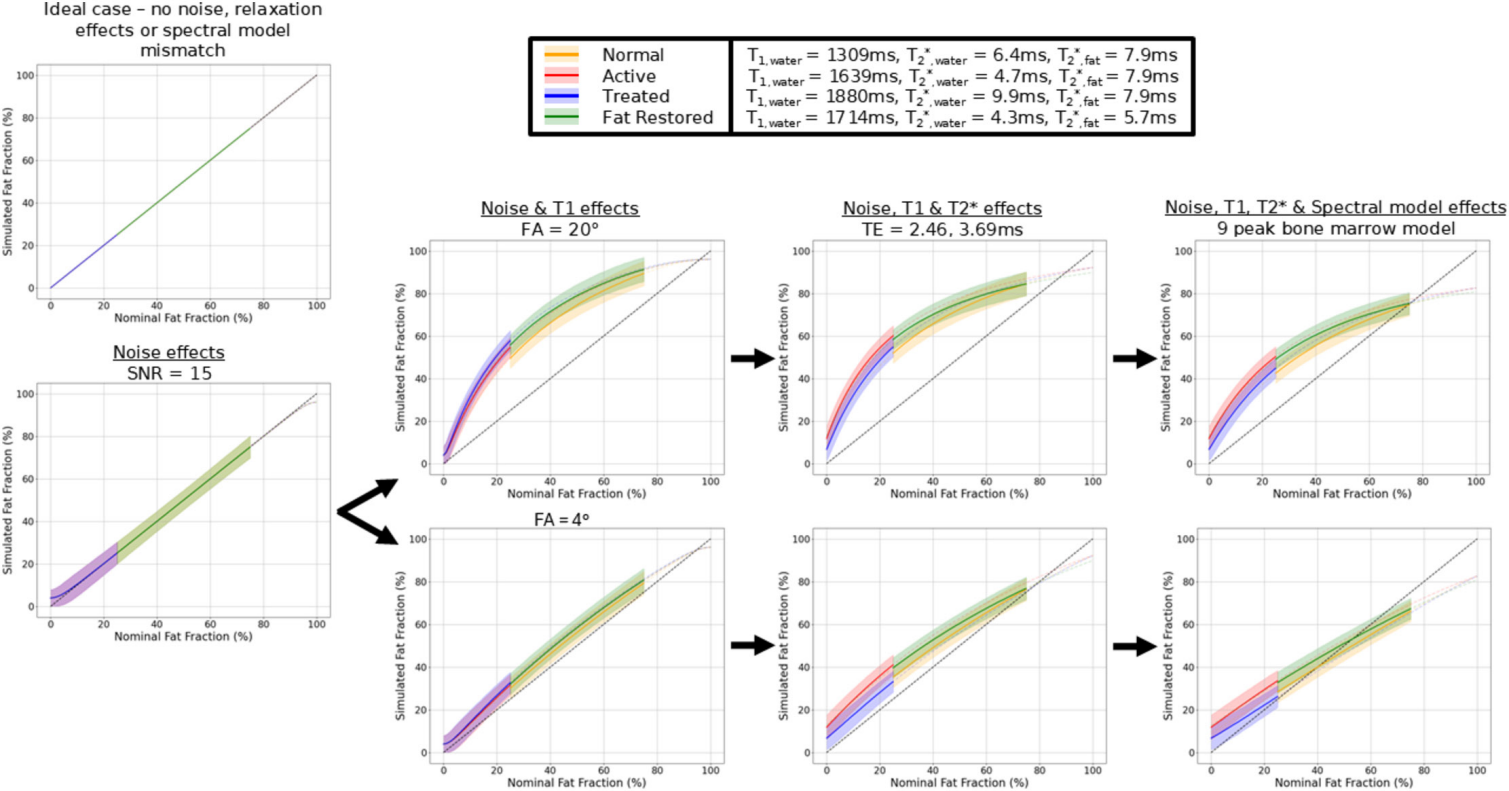
Monte–Carlo simulation of fat‐water separation with relaxation times from this study. Averaged measured fat fractions are shown at each fat fraction value on a 1:1:100 grid. Simulation parameters: *T*_1,fat_ = 370 ms, TR = 7.14 ms, TE = 2.48, 3.69 ms, 10,000 iterations for each fat fraction. All values for water and fat (methylene) T1 and T2* are based on subgroup averages reported in this study. The normal‐appearing T2*_methylene_ was used in active and treated lesions. Bolded colours indicate plausible regions for each disease state. Shaded regions demonstrate the 95% confidence interval.

Simulations demonstrate the impact of noise, relaxation time biases and spectral model in various bone marrow disease states for 2‐point FF estimates in a compounding fashion (Figure 8). The inclusion of PD‐weighting with a low flip angle results in a reduction in T1 bias, although this is not completely eliminated. The T2* difference between water and fat, particularly in active and treated disease, results in a step change in FF. Irrespective of flip angle, the extremes in the FF range are sensitive to noise bias.

## 4 Discussion

In this preliminary study, we have demonstrated that water relaxation times vary significantly between bone lesions and normal‐appearing marrow in the WB‐MR patient population. All parameters reported, including MRS‐PDFF, were highly repeatable (ICC > 0.95, CoV < 10%), although several fat peaks were not reliably resolvable at 3T for fitting. This was overcome by constraining several fat peaks to share a relaxation time with methylene. Relaxation times of modelled fat peaks showed statistically insignificant variation amongst disease states and were similar to those previously reported in adipose tissue and bone marrow at 3T [27, 28].

The high repeatability of fat quantification using MRS achieved in this study is comparable to Li et al., where a coefficient of variation of 1.7% was reported in the lumbar spine [31]. Neumayer et al. also measured the repeatability of the T1 of the water and methylene peaks using STEAM with a varying repetition time, reporting a lower coefficient of variation (2.0% for water and 3.0% for methylene) than in this study [32]. The results in this study consider repeatability across all modelled peaks and are derived from a smaller sample size, potentially explaining the discrepancy alongside choices in measurement approach. Finally, the statistically significant bias in R2* of the femoral head from repeatability analysis could be due to the small sample size or the heterogeneity of the structure resulting in high sensitivity to the choice of position during localization.

The observed variation in relaxation time with disease state reflects alterations to both the composition and microenvironment of the marrow. Water T1 and T2 can be considered correlates of the ADC, which has demonstrated significant diagnostic value in WB‐MRI. With treatment, bone lesions become necrotic due to widespread cell death, leading to lengthening of relaxation times and ADC. Conversely, resolution of a lesion due to increasing fat content can be accompanied by an osteoblastic response and fibrosis, which could explain the elevated R2* in fat‐restored lesions compared to healthy and normal‐appearing marrow [33].

The water T1 and R2* relaxation times presented in this study have significant implications for the interpretation of signal‐weighted FFs reported in clinical studies [4, 5, 34, 35]. While both PD‐ and T1‐weighted 2‐point Dixons are monotonically increasing with the true FF, the effect of T1 bias at larger flip angles results in the measured FF always being an overestimation, particularly with low fat content. At worst, this overestimation can exceed 30%. It is possible for the FF of a lesion measured with T1‐weighted 2‐point Dixon acquisitions to have the appearance of being fat restored where there has been no actual change in fat content, particularly in the case of a noisy measurement. This simulated result is similar to in vivo data presented in a recent imaging study comparing 2‐point Dixon methods to PD‐weighted multiecho Dixon imaging [35].

While low flip angles mitigate the T1‐related bias in 2‐point FF, they may not prove sufficient for a fully quantitative FF using imaging in bone marrow due to residual T1 bias. Working at 1.5 T is conventional for WB‐MRI and would be beneficial for fat quantification in bone marrow owing to the prolonged T2* and possibly weaker T1 disparity between water and fat [36]. Furthermore, the SNR efficiency of fat‐water separation has a strong dependence on choice in echo times and number of echoes, which can be more flexibly achieved at 1.5 T due to the slower phase cycling of fat relative to water [37]. To overcome the residual T1 bias, variable flip angle chemical shift encoded MRI with a complex‐based reconstruction, as demonstrated in recent studies for liver [38, 39], would likely be a step towards improving quantitative accuracy of FF in malignant bone disease and assessment of T1 of water as a potential imaging biomarker. Considerations will also need to be made regarding the biexponential behaviour of T2* in bone marrow suggested by the differences seen in the R2* of water and fat. A correction method has also been reported in a previous study, which showed that T2′ correction based on population averaged T2 values in water and fat can be superior to dual‐T2* fitting with regards to noise performance of fat quantification in vertebral bone marrow [9].

This study has several limitations. Foremost, the small sample size and its single‐centre and single‐scanner nature limit generalizability. There is also no consideration of relaxation times between osteoblastic and osteolytic disease. As a result, the inferential statistics used in this study should be viewed with caution.

A further limitation is the poor spectral separation achievable at 3T in bone marrow, which prevents reliable separation of weaker or crowded fat resonances. Future work may employ developments shown by Afkham et al. for improving the shim [40], yet despite the potential deficiencies of B_0_ shimming, linewidths reported in this study are largely comparable with other studies conducted at 3T in bone marrow [9, 41]. Fat linewidths were not uniquely modelled, which could be a source of error owing to many fat peaks having contributions from multiple locations in the triglyceride and different J‐coupling behaviour.

The scatter of the data between R2*, derived from linewidths in MRS, and imaging could reflect that the imaging method, working at the voxel‐scale, is capturing physiological susceptibility variations while the MRS estimates are limited by shim. There is also the possibility of the mismatch in spectral model of fat (liver used over bone marrow in the imaging method), which has been demonstrated to be a source of bias in R2* [42]. There is also the fact that the imaging method reports an effective R2*, which is shared between fat and water.

Both the MRS‐PDFF and MRI‐PDFF values reported are also likely to have some residual T1 bias. This is due to incomplete longitudinal relaxation of the water peak during the TR of the variable echo time data and the choice of flip angle, respectively. The TRs employed in this study were a compromise to prioritize acquiring finely sampled relaxometry data in the limited time following the patients' clinical examinations. However, rapid techniques for combined relaxometry and fat quantification have been developed, typically involving sampling spectra in a two‐dimensional space of TE and TR or TI with minimal averaging [43, 44, 45]. These were not explored, although future work evaluating these approaches would be clinically valuable, particularly with the provided data here available to optimize spectral sampling. Finally, the imaging method used for PDFF estimation is a hybrid approach that finishes with magnitude‐based fitting in its pipeline. Magnitude fitting has inferior noise performance compared to complex‐based approaches and potential for swaps at FFs between 40% and 60%, which are common in bone marrow applications [46].

## 5 Conclusion

In this study, we report MRS‐based relaxation time measurements for healthy bone marrow and bone lesions. The relaxation times measured in this study result in T1‐ and T2*‐related biases in bone lesion FF estimated using 2‐point T1‐weighted Dixon, as recommended in international guidelines, compared to MRS at 3T. Our results suggest that a quantitative imaging approach, such as a vendor product providing PDFF, will provide improved agreement with MRS. The relaxation time variance seen in the population suggests the need for careful consideration of T1 and T2* related biases in imaging‐based fat quantification methods applied in malignant bone disease. Although these relaxation time variations are problematic for fat quantification, they may also prove to be potential imaging biomarkers for treatment response in malignant bone disease. These data will be useful for other researchers developing imaging techniques in malignant bone disease.

## Author Contributions

**Yassine N. Azma, David J. Collins, Christina Messiou, Dow‐Mu Koh, Nina Tunariu, Jessica M. Winfield:** conceptualization. **Yassine N. Azma, Pete J. Lally, David J. Collins, Jessica M. Winfield:** methodology. **Yassine N. Azma:** software. **Yassine N. Azma, Jessica M. Winfield:** validation. **Yassine N. Azma, Jessica M. Winfield:** formal analysis. **Yassine N. Azma, David J. Collins, Jessica M. Winfield:** investigation. **Yassine N. Azma:** data curation. **Yassine N. Azma:** writing – original draft. **All:** writing – review and editing. **Yassine N. Azma:** visualization. **Pete J. Lally, Christina Triantafyllou, Neal K. Bangerter, Jessica M. Winfield:** supervision. **Yassine N. Azma, Pete J. Lally, Neal K. Bangerter, Christina Triantafyllou, Jessica M. Winfield:** project administration. **Christina Messiou, Dow‐Mu Koh, Nina Tunariu, Christina Triantafyllou, Jessica M. Winfield:** funding acquisition.

## Funding

This work was supported by the National Institute for Health and Care Research (NIHR203314), Siemens Healthineers (230151) and Cancer Research UK (28677).

## Conflicts of Interest

Christina Triantafyllou is an employee of Siemens Healthineers. Yassine N. Azma receives PhD studentship funding from Siemens Healthineers.

## Supporting information


**Figure S1:** Nonselective hyperbolic secant pulse for inversion.
**Figure S2:** Frequency profiles in Mxy and Mz without relaxation and with T2* = 5 ms.
**Figure S3:** Selective Hamming‐sinc pulse for excitation.
**Figure S4:** Magnetization dependence with isochromat frequency and position for an excitation performed at the centre frequency. The histogram depicts the distribution of magnetization in the region of interest.
**Figure S5:** Magnetization dependence with isochromat frequency and position for an excitation performed at −2.3 ppm. The histogram depicts the distribution of magnetization in the region of interest.
**Table S1:** Pooled coefficient of variation per resonance for relaxation times. The R2* coefficient of variation for methylene is separate to all other fat peaks. For T2, the coefficient of variation for methylene is shared with all other fat peaks that are not composite.
**Table S2:** Per‐subject average coefficient of variation per resonance for relaxation times. The R2* coefficient of variation for methylene is separate to all other fat peaks. For T2, the coefficient of variation for methylene is shared with all other fat peaks that are not composite.
**Figure S6:** Comparison of joint fitting all multi‐TE data and only TE20–TE50 for the T2 relaxation times of methylene (representing all fat peaks that are not fitted separately), composite (the superposition of peaks at 2.03 and 2.25 ppm) and water.
**Figure S7:** Comparison of calculating PDFF with all multi‐TE data and only TE20–TE50.

## Data Availability

The data that support the findings of this study are available from the corresponding author upon reasonable request.
