# Supplementary material for: Patient‐Specific Biases in Fat Fraction Estimates of Malignant Bone Marrow due to Relaxation Times Measured With STEAM at 3T
Source: NMR Biomed. 2026 Feb 24;39(4):e70242. doi: 10.1002/nbm.70242 (PMC12932037; doi:10.1002/nbm.70242)
Supplement: Supplementary file 1 — Figure S1: Nonselective hyperbolic secant pulse for inversion. Figure S2: Frequency profiles in Mxy and Mz without relaxation and with T2* = 5 ms. Figure S3: Selective Hamming‐sinc pulse for excitation. Figure S4: Magnetization dependence with isochromat frequency and position for an excitation performed at the centre frequency. The histogram depicts the distribution of magnetization in the region of interest. Figure S5: Magnetization dependence with isochromat frequency and position for an excitation performed at −2.3 ppm. The histogram depicts the distribution of magnetization in the region of interest. Table S1: Pooled coefficient of variation per resonance for relaxation times. The R2* coefficient of variation for methylene is separate to all other fat peaks. For T2, the coefficient of variation for methylene is shared with all other fat peaks that are not composite. Table S2: Per‐subject average coefficient of variation per resonance for relaxation times. The R2* coefficient of variation for methylene is separate to all other fat peaks. For T2, the coefficient of variation for methylene is shared with all other fat peaks that are not composite. Figure S6: Comparison of joint fitting all multi‐TE data and only TE20–TE50 for the T2 relaxation times of methylene (representing all fat peaks that are not fitted separately), composite (the superposition of peaks at 2.03 and 2.25 ppm) and water. Figure S7: Comparison of calculating PDFF with all multi‐TE data and only TE20–TE50. [file NBM-39-e70242-s001.docx]

# Patient-specific biases in fat fraction estimates of malignant bone marrow due to relaxation times measured with STEAM at 3T

## Supplementary Materials

### Inversion Pulse Design

Owing to the short T2* of bone marrow at 3T, a relatively short duration was chosen for the hyperbolic secant inversion pulse. The parameters of the pulse were μ = 5, BW = 2400Hz, T = 5000ms, and the pulse’s magnitude and frequency sweep are defined as stated in Equations 1-3.

$$\begin{aligned} \beta=\frac{\pi BW}{\mu} \#1 \end{aligned}$$

$$\begin{aligned} A\left( t \right)=\mathrm{sech} \left( \beta\left( t-\frac{T}{2} \right) \right)\#2 \end{aligned}$$

$\begin{aligned} F\left( t \right)= -\mu\beta\tanh\left( \beta\left( t-\frac{T}{2} \right) \right) \#3 \end{aligned}$

The pulse magnitude (to achieve inversion) and phase are displayed below in Figure 1.


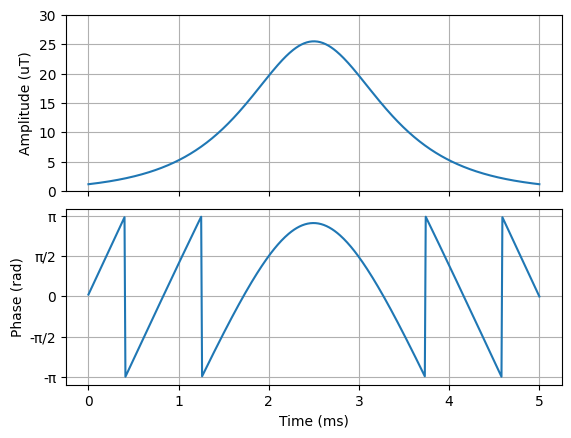


Figure 1: Non-selective hyperbolic secant pulse for inversion

The result of a Bloch simulation is shown below in Figure 2 without relaxation and a time step of 10us. The loss in inversion efficiency due to T2* relaxation during the inversion pulse motivates the need for an inversion efficiency correction.


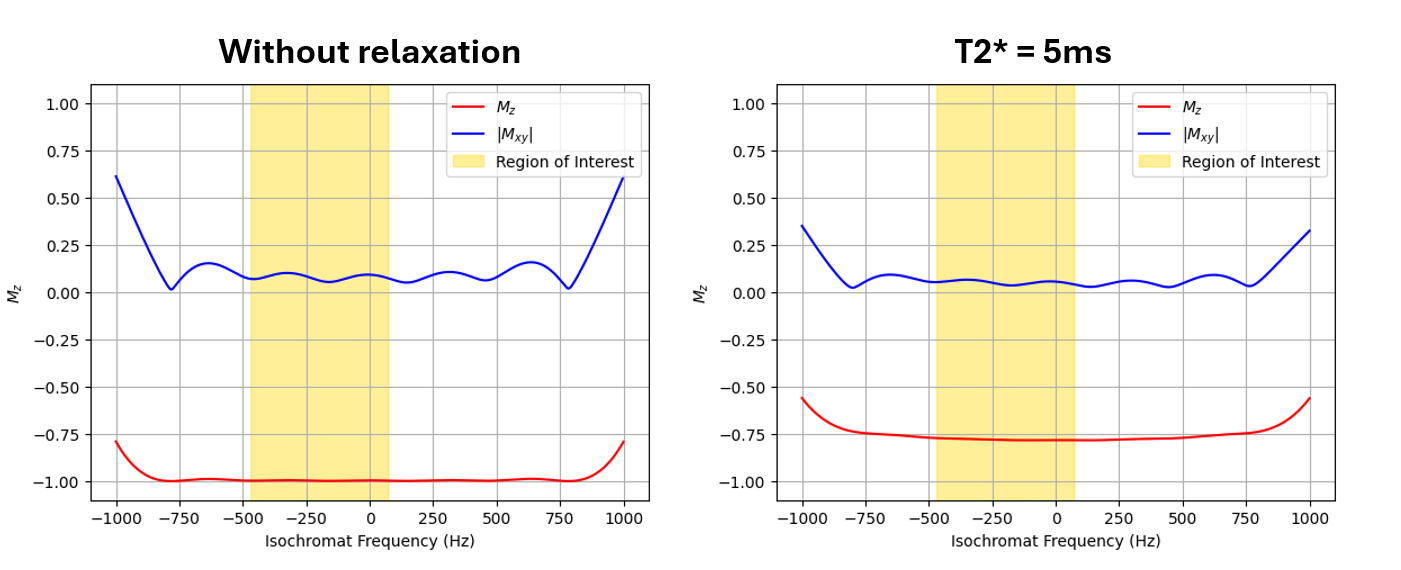


Figure 2: Frequency profiles in M_xy_ and M_z_ without relaxation and with T2* = 5ms.

Post-inversion spoiler gradients were included (45.5mT/m⋅ms gradient moment along a single axis) and played along all gradient axes. This results in a theoretical minimum phase twist of 185 rad across the smallest dimension of a voxel used in the study (15mm), which far exceeds the condition for strong gradient spoiling.

### Chemical Shift Displacement

The excitation pulse used in all experiments was a Hamming-filtered sinc with the following parameters: BW = 3600Hz, T = 2.4ms.

The pulse magnitude (to achieve a 90 degree excitation) and phase are displayed below.


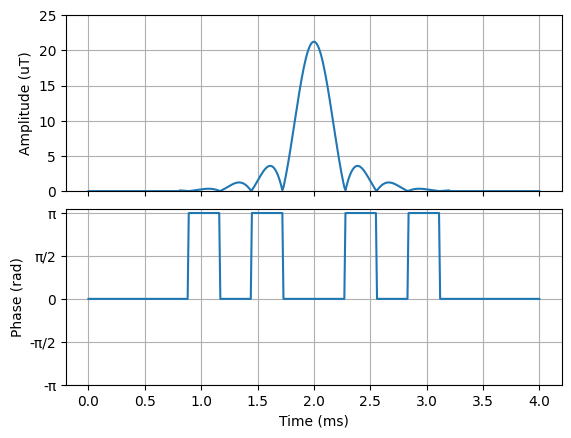


Figure 3: Selective Hamming-sinc pulse for excitation

$$\begin{aligned} A\left( t \right)=H\left( t \right)*\mathrm{sinc} \left( BW\left( t-\frac{T}{2} \right) \right) \#4 \end{aligned}$$

$$\begin{aligned} H\left( t \right)=0.54-0.46\cos\left( \frac{2\pi t}{T} \right) \#5 \end{aligned}$$

The impacts of chemical shift displacement were assessed through Bloch simulation with a timestep of 10us. The gradient amplitude was 4.28mT/m, which is sufficient for a 20mm voxel. The frequency resolution was 2Hz (1001 unique frequencies), spatial resolution of 0.1mm (201 unique positions on the z axis). Results of these simulations can be seen in Figures 4 and 5.


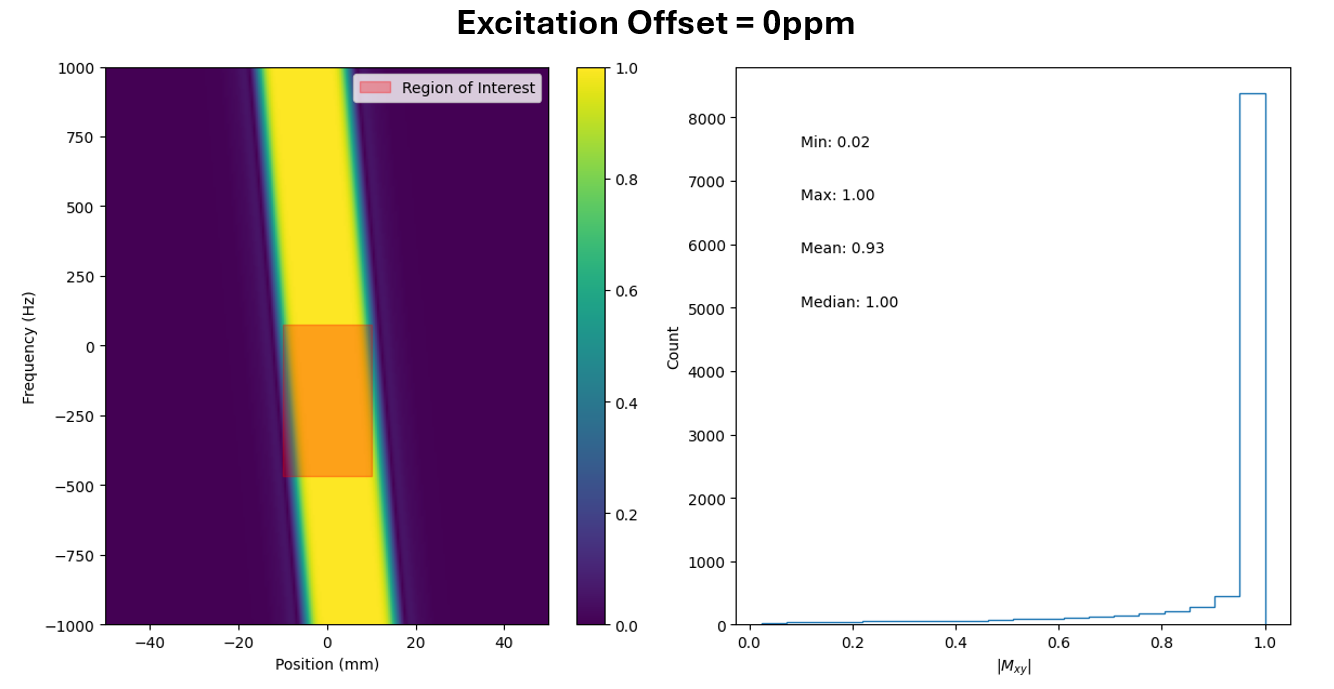


Figure 4: Magnetization dependence with isochromat frequency and position for an excitation performed at the centre frequency. The histogram depicts the distribution of magnetization in the region of interest.


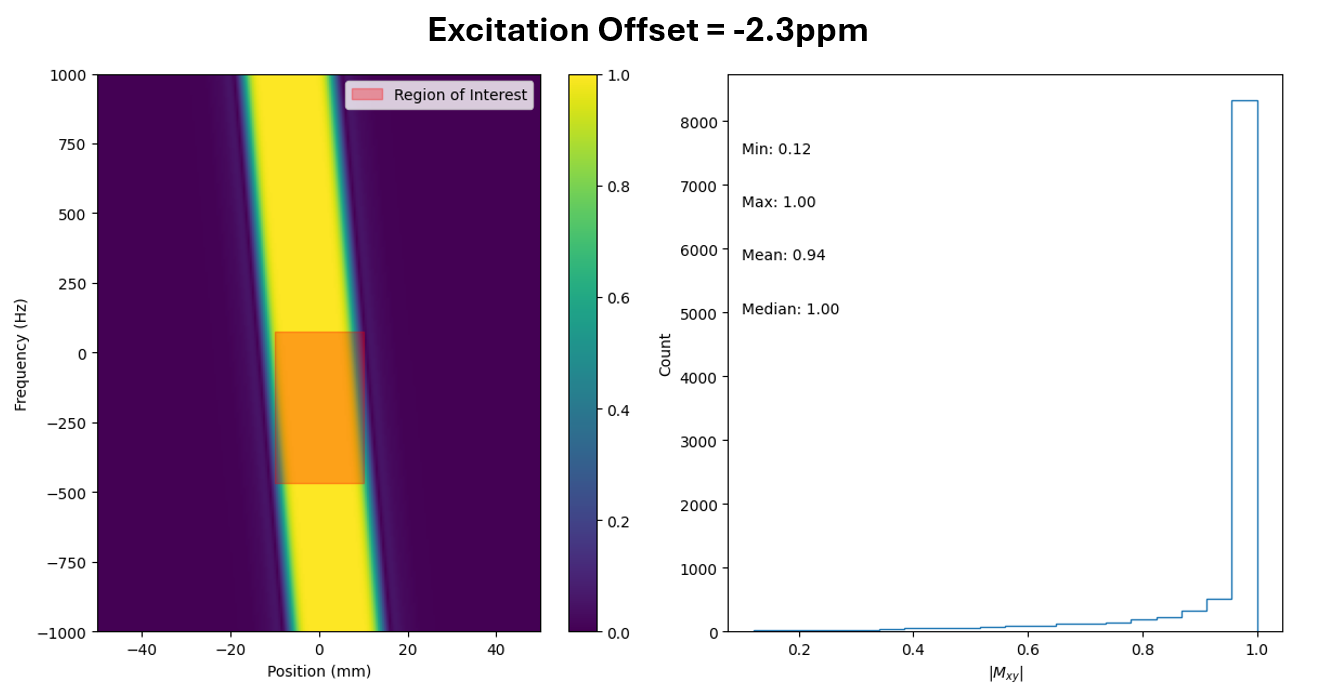


Figure 5: Magnetization dependence with isochromat frequency and position for an excitation performed at -2.3ppm. The histogram depicts the distribution of magnetization in the region of interest.

Reduction in chemical shift displacement in the magnetization region of interest was minimal with different excitation offset frequencies at 3T for the given excitation pulse.

### Pooled Coefficient of Variation per Resonance in Volunteers – Relaxation Times

| Resonance | L5  (N = 5) | | | Femoral Head (N = 5) | | | | |
| --- | --- | --- | --- | --- | --- | --- | --- | --- |
|  | T1  (%) | T2  (%) | R2* (%) | | T1  (%) | T2 (%) | R2* (%) |  |
| Methyl (0.9ppm) | 9.0 | N/A | N/A | | 15.8 | N/A | N/A |  |
| Methylene  (1.3ppm) | 5.9 | 4.5 | 8.4 | | 3.3 | 4.4 | 7.6 |  |
| Composite  (2.05ppm) | 6.2 | 10.4 | N/A | | 3.9 | 6.3 | N/A |  |
| Diacyl (2.77ppm) | 10.2 | N/A | N/A | | 10.2 | N/A | N/A |  |
| Water  (4.7ppm) | 10.7 | 14.0 | 10.4 | | 5.2 | 21.4 | 12.6 |  |
| Olefin  (5.31ppm) | 26.1 | N/A | N/A | | 15.6 | N/A | N/A |  |

Table 1: Pooled coefficient of variation per resonance for relaxation times. The R2* coefficient of variation for methylene is separate to all other fat peaks. For T2, the coefficient of variation for methylene is shared with all other fat peaks that are not composite.

### Per-Subject Average Coefficient of Variation per Resonance in Volunteers – Relaxation Times

| Resonance | L5  (N = 5) | | | Femoral Head (N = 5) | | | | |
| --- | --- | --- | --- | --- | --- | --- | --- | --- |
|  | T1  (%) | T2  (%) | R2* (%) | | T1  (%) | T2 (%) | R2* (%) |  |
| Methyl (0.9ppm) | 8.9 | N/A | N/A | | 15.8 | N/A | N/A |  |
| Methylene  (1.3ppm) | 5.9 | 3.8 | 8.4 | | 3.1 | 4.1 | 7.5 |  |
| Composite  (2.05ppm) | 6.2 | 10.4 | N/A | | 3.7 | 6.2 | N/A |  |
| Diacyl (2.77ppm) | 7.9 | N/A | N/A | | 10.0 | N/A | N/A |  |
| Water  (4.7ppm) | 10.7 | 13.8 | 10.3 | | 5.1 | 21.3 | 12.5 |  |
| Olefin  (5.31ppm) | 9.0 | N/A | N/A | | 15.2 | N/A | N/A |  |

Table 2: Per-subject average coefficient of variation per resonance for relaxation times. The R2* coefficient of variation for methylene is separate to all other fat peaks. For T2, the coefficient of variation for methylene is shared with all other fat peaks that are not composite.

### Effects of J-Coupling on T2 and PDFF

At long echo times, fat resonances are subject to J-coupling, which can confound the estimation of T2 and PDFF. The healthy L5 and the entirety of the patient cohort were subject to multi-TE joint fitting with a subset of the echo times (from TE=20ms to TE=50ms) and with all the echo times. A comparison of T2 and PDFF estimation with the subset and the full multi-echo data are shown below in Figures 6 and 7.


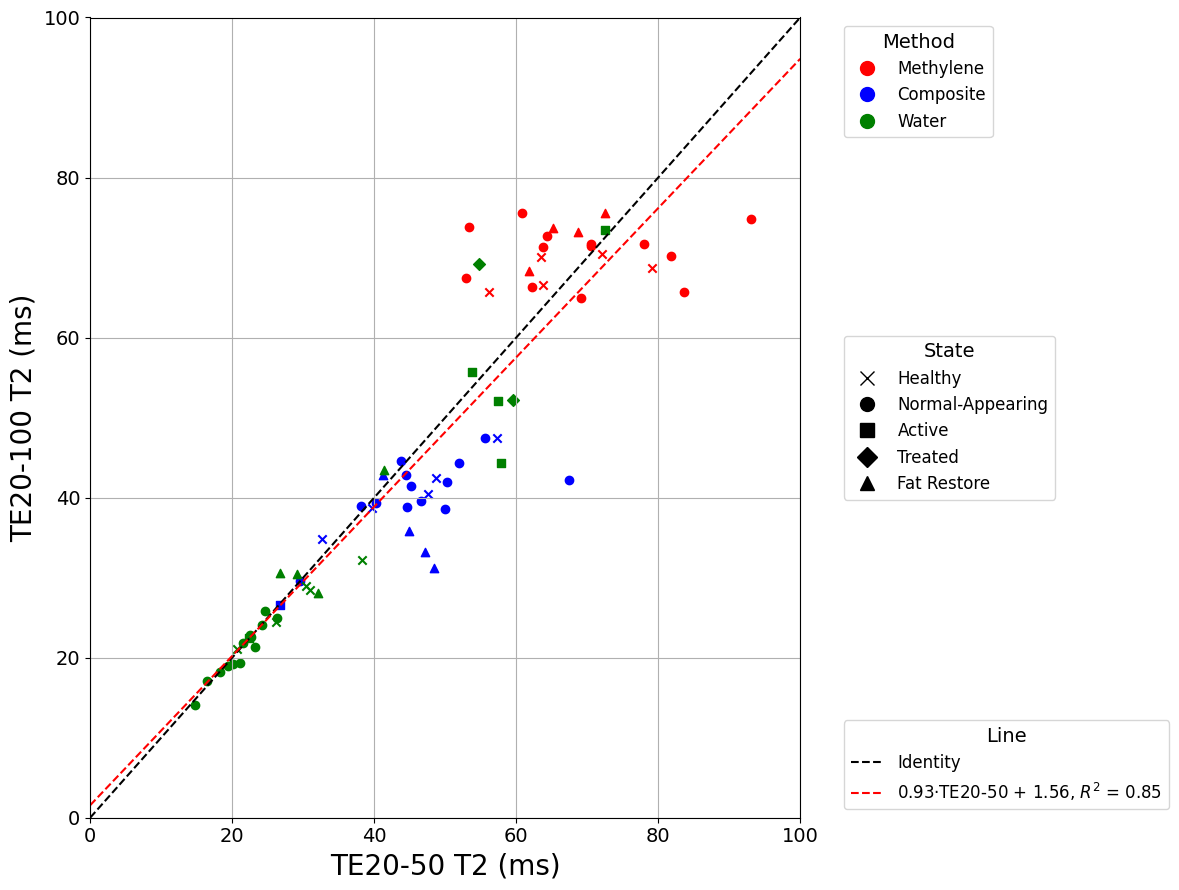


Figure 6: Comparison of joint fitting all multi-TE data and only TE20 – TE50 for the T2 relaxation times of methylene (representing all fat peaks that are not fitted separately), composite (the superposition of peaks at 2.03 and 2.25ppm) and water.

###
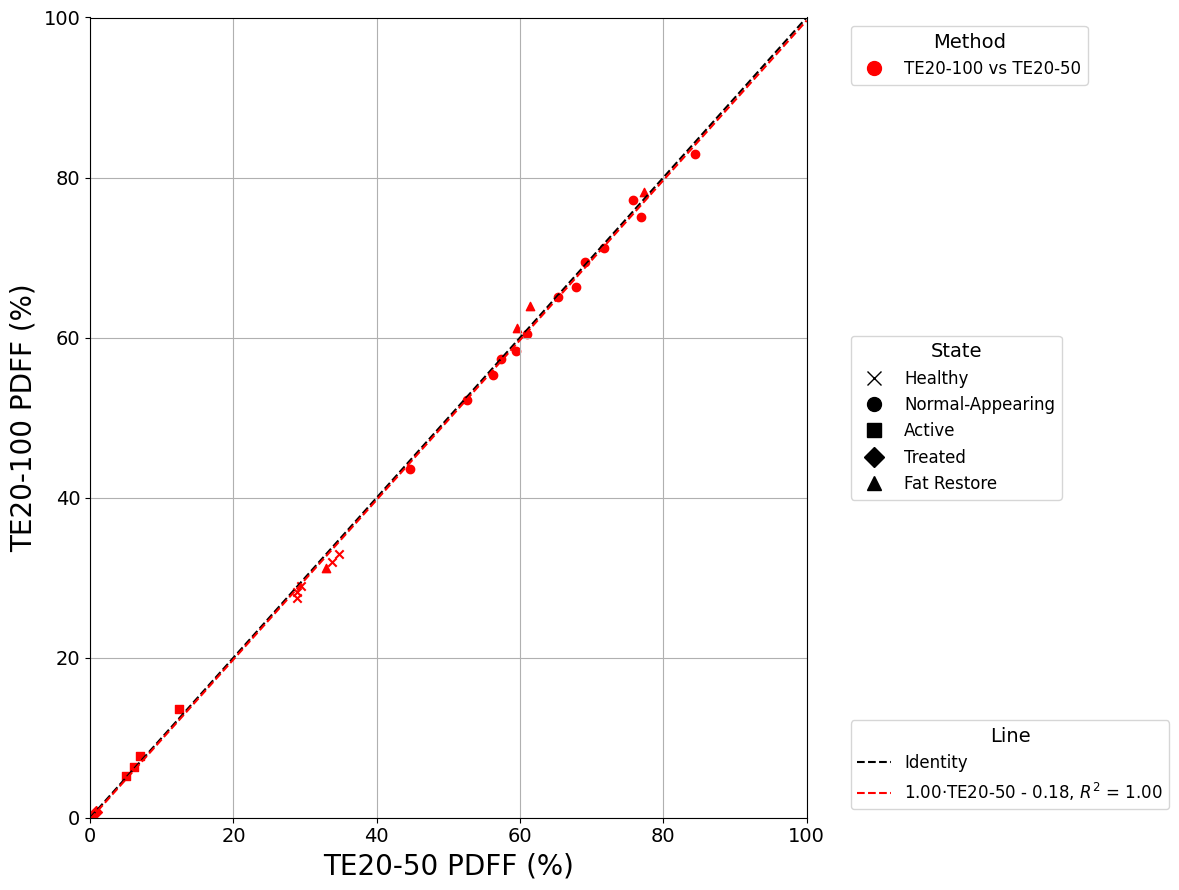


Figure 7: Comparison of calculating PDFF with all multi-TE data and only TE20 – TE50.
